# Clinical significance of the hemoglobin-to-red blood cell distribution width ratio in patients with severe OSAS

**DOI:** 10.1038/s41598-025-96693-1

**Published:** 2025-04-12

**Authors:** Ni Tan, Yuan He, Guangcai Li, Peijun Liu

**Affiliations:** 1 Department of Respiratory and Critical Care Medicine, The Central Hospital of Enshi Tujia and Miao Autonomous Prefecture, Enshi, China; 2 Department of Ultrasonography, The Central Hospital of Enshi Tujia and Miao Autonomous Prefecture, Enshi, China

**Keywords:** OSAS, AHI, Predictive biomarkers, HRR, ROC, Neuromuscular disease, Sleep disorders

## Abstract

This study aimed to evaluate the clinical significance of the hemoglobin-to-red blood cell distribution width ratio (HRR) in patients with severe obstructive sleep apnea syndrome (OSAS). A retrospective analysis was conducted on 216 patients who underwent polysomnography (PSG) between January 2018 and February 2023. Patients were divided into two groups according to the apnea-hypopnea index (AHI): severe OSAS (*n* = 131) and non-severe OSAS (*n* = 85). Comparisons were performed between the two groups regarding baseline characteristics, AHI, minimum blood oxygen saturation (MinSaO₂), and hematological parameters including white blood cell (WBC) count, red blood cell (RBC) count, platelet count (PLT), hemoglobin (Hb), red blood cell distribution width (RDW), hematocrit (HCT), mean corpuscular volume (MCV), mean corpuscular hemoglobin (MCH), mean corpuscular hemoglobin concentration (MCHC), and HRR. Correlation analyses were conducted to assess relationships between AHI, MinSaO₂, and HRR in the severe OSAS group. Receiver operating characteristic (ROC) curve analysis revealed that HRR had an area under the curve (AUC) of 0.84 for predicting severe OSAS, with a sensitivity of 81.68%, specificity of 72.94%, and 95% confidence interval (CI) of 0.784–0.886. The levels of AHI, MinSaO₂, Hb, and HRR were significantly higher in the severe OSAS group. Elevated AHI levels may indicate an increased risk of severe OSAS. HRR may serve as an early predictive biomarker for severe OSAS, facilitating timely intervention and potentially reducing complication risks.

**Supplementary Information:**

The online version contains supplementary material available at 10.1038/s41598-025-96693-1.

## Introduction

Obstructive sleep apnea syndrome (OSAS) is the most common chronic disease of the respiratory system encountered in clinical practice during sleep. It is mainly characterized by intermittent hypoxemia, hypercapnia, and disturbances in sleep architecture^1^. Clinically, it presents with symptoms such as daytime sleepiness, snoring during sleep, observed episodes of apnea, and interruptions in breathing, which can further lead to impaired functions in target organs and pose significant health risks to patients^2,3^. In recent years, with the improvement of living standards and environments, the incidence of OSAS has gradually increased. However, many patients do not pay adequate attention to their condition, and it is often at a severe stage by the time they seek medical attention^4^.

The pathogenesis of OSAS remains unclear and requires further study. Diagnosis is typically based on clinical signs, symptoms, complications, and sleep monitoring. Polysomnography (PSG) is the current gold standard, but it is time-consuming, complex, and often poorly tolerated by patients. Factors like sleep quality and sensor placement can also affect the accuracy of results. Therefore, developing a more convenient and reliable diagnostic method for severe OSAS is a key focus of current research^5^.

Recent studies have identified red blood cell distribution width (RDW) as a potential marker of inflammation in subclinical settings^6–8^. RDW, a routine blood test indicator associated with chronic hypoxia, measures the variability in red blood cell size in peripheral circulation. Increased erythrocyte destruction or ineffective erythropoiesis leads to greater variability in cell size, reflected in higher RDW, which is linked to inflammation and vascular endothelial injury. Hemoglobin (Hb), another routine blood test indicator, reflects anemia. Studies on organ damage in OSAS have shown a significant correlation between intermittent hypoxia and increased Hb levels in OSAS^9^. Additionally, the hemoglobin-to-red blood cell distribution width ratio (HRR) has been associated with prognosis in patients with cancers, decompensated cirrhosis, lymphoma, and rheumatoid arthritis^10,11^. However, no studies have examined HRR levels in OSAS patients. This study aims to explore HRR as a predictor by analyzing routine blood parameters in OSAS patients and assessing changes in HRR, to provide a reference for diagnosing and treating severe OSAS.

## Materials and methods

### Study design

This retrospective study included 216 patients diagnosed with OSAS via PSG in the departments of respiratory and critical care medicine and otolaryngology-head and eck surgery at our hospital between January 2018 and February 2023. The study cohort consisted of 160 male and 56 female patients, aged 18 to 80 years, with a body mass index (BMI) ranging from 20 to 38 kg/m². Due to the retrospective nature of the study, the need to obtain the informed consent was waived by the ethical committees of central hospital of enshi tujia and miao autonomous prefecture. All methods were performed in accordance with relevant guidelines and regulations, and all experimental protocols were approved by the same ethics committee.

### Inclusion and exclusion criteria

The inclusion criteria for this study were patients diagnosed with OSAS by PSG. The exclusion criteria were as follows: patients with other respiratory diseases, those with severe heart, liver, or kidney disease, patients with hematological diseases or malignant tumors, individuals with a history of alcoholism, and those taking long-term sedative medications.

### Group

Based on their Apnea-Hypopnea Index (AHI), the study participants were divided into two groups, following the 2018 multidisciplinary guidelines for the management of obstructive sleep apnea in adults. The control group included 85 patients with an AHI < 30, while the experimental group consisted of 131 patients with an AHI ≥ 30.

### Measurements

Body Mass Index (BMI) was calculated for all participants. PSG was conducted overnight, with participants abstaining from alcohol, tea, coffee, tobacco, and medications affecting sleep, breathing, or heart rate before the test. During PSG, parameters such as oral and nasal airflow, chest and abdominal movements, body position, snoring, electroencephalogram (EEG), and minimum blood oxygen saturation (MinSaO_2_) were recorded. The AHI and MinSaO_2_ values were analyzed both by computer and manually corrected. Blood samples were collected in the morning immediately after overnight PSG, following a fasting period of no more than 12 h. In addition, routine blood tests were performed to measure white blood cells (WBC), red blood cells (RBC), platelets (PLT), hemoglobin (Hb), RDW, hematocrit (HCT), mean corpuscular volume (MCV), mean corpuscular hemoglobin (MCH), and mean corpuscular hemoglobin concentration (MCHC). The HRR was then calculated from these parameters.

### Logistic regression analysis

A multivariate logistic regression analysis was conducted to assess the association between key clinical and hematological parameters and the risk of developing severe OSAS. Independent variables included AHI, MinSaO₂, RBC, Hb, and HRR. The model calculated odds ratios (ORs) and 95% confidence intervals (CIs) to estimate the relative risk for each variable. Statistical significance was defined as *P* < 0.05. All analyses were performed using R software (version 4.2.0), with the statistics package for logistic regression modeling and the pROC package for performance evaluation. Standardization of continuous variables was applied where necessary to ensure interpretability and consistency.

### Pearson correlation analysis

Pearson correlation analysis was conducted using R software (version 4.2.0). The statistics package was utilized for the calculation of Pearson’s correlation coefficients to examine the linear relationships between AHI, MinSaO₂, and HRR in severe OSAS patients. This method evaluates the strength and direction of the linear association between two continuous variables. The correlation coefficient () ranges from − 1 to 1, with positive values indicating a positive correlation, negative values indicating a negative correlation, and values near 0 suggesting no linear association. Statistical significance was determined at *P* < 0.05, and the results were visualized using the ggplot2 package.

### Receiver operating characteristic (ROC) curve analysis

ROC curve analysis was performed in R using the pROC package to assess the diagnostic performance of HRR in predicting severe OSAS. The area under the curve (AUC) was calculated to measure the overall accuracy of HRR as a diagnostic marker, with values closer to 1 indicating better discriminatory ability. Sensitivity and specificity values at various HRR thresholds were determined, and the optimal cutoff value was selected using the Youden index, defined as(Sensitivity + Specificity − 1). The ROC curve and AUC were visualized using the ggplot2 package for clear graphical representation.

### Statistical analysis

This study used SPSS version 24.0 for statistical analysis. Normally distributed continuous variables were reported as mean ± standard deviation and compared using the independent t-test, while categorical variables were expressed as frequencies and percentages and analyzed with the chi-square test. One-way ANOVA was used for comparisons across multiple groups.

## Results

### Baseline characteristics and their association with OSAS severity

There were no statistically significant differences in age between the two groups of study participants. However, BMI and male sex were significantly associated with the severity of OSAS, as evidenced by their differences between the two groups (*P* < 0.05, Table 1). These findings suggest that BMI and male sex may play important roles in determining OSAS severity.

**Table 1:** Comparison of baseline characteristics.

Variables	Non-severe OSAS	Severe OSAS	t	*P*
Age(years)	51.55 ± 12.57	49.7 ± 13.88	0.993	0.322
Male(n%)	53(62.4)	107(81.7)		0.002
BMI(Kg/m^2^)	25.58 ± 5.43	26.57 ± 2.75	− 2.703	< 0.001

### OSAS parameters and blood indices

Following the findings presented in Table 1, further analysis showed significant differences in AHI, MinSaO₂, RBC, Hb, HCT, MCH, MCHC, and HRR levels between the two groups, indicating that these parameters are closely associated with the severity of OSAS (*P* < 0.05, Table 2). However, no statistically significant differences were found in WBC, PLT, RDW, or MCV levels between the two groups, suggesting that these indices may have limited relevance to the severity of OSAS (*P* > 0.05, Table 2).A detailed comparison of OSAS-related parameters and routine blood indices between the groups is provided in Table 2, highlighting the significant findings while identifying parameters that showed no apparent relationship with disease severity.

**Table 2:** Comparison of OSAS parameters and routine blood indexes between the two groups.

Variables	non-severe OSAS	severe OSAS	t	*P*
AHI	16.50 ± 18.14	58.58 ± 21.63	− 15.419	< 0.001*
MinSaO_2_(%)	83.72 ± 5.69	69.32 ± 11.15	12.480	< 0.001*
WBC(10^9^/L)	6.44 ± 1.97	6.58 ± 1.89	− 0.518	0.605
RBC (10^12^/L)	4.58 ± 0.64	4.89 ± 0.75	− 3.101	0.002*
PLT(10^9^/L)	193.54 ± 47.36	200.27 ± 56.75	− 0.907	0.366
Hb(g/L)	137.95 ± 21.31	149.63 ± 20.71	− 4.003	< 0.001*
RDW(%)	13.36 ± 1.26	13.36 ± 1.16	− 0.045	0.964
HCT(%)	0.41 ± 0.06	0.43 ± 0.06	− 4.351	0.106
MCV(fl.)	91.65 ± 6.62	92.72 ± 5.64	− 1.272	0.205
MCH(pg)	30.20 ± 2.55	30.45 ± 2.09	− 2.049	0.142
MCHC(g/L)	329.55 ± 11.81	332.70 ± 11.07	− 1.989	0.248
HRR	10.46 ± 2.06	11.29 ± 1.81	− 3.116	0.002*

**Abbreviations**: AHI, apnea hypopnea index; MinSaO_2_, minimum blood oxygen saturation; WBC, blood white blood cell; RBC, red blood cell; PLT, platelet; Hb, hemoglobin; RDW, red blood cell distribution width; HCT, hematocrit; MCV, mean corpuscular volume; MCH, mean corpuscular hemoglobin; MCHC, mean corpuscular hemoglobin concentration; HRR, hemoglobin to red blood cell distribution width ratio.

### **Significant Predictors of Severe OSAS**

Building on the findings presented in Table 2, logistic regression analysis further explored the association between key OSAS parameters and routine blood indices with the risk of developing severe OSAS(Table 3). The logistic regression analysis revealed that AHI, MinSaO₂, Hb, and HRR were significantly associated with the risk of severe OSAS (*P* < 0.05). AHI (OR = 1.29,95%CI = 1.14 − 1.44) and HRR (OR = 1.09,95%CI = 1.05 − 1.16) exhibited strong positive associations with severe OSAS, while MinSaO₂ was inversely correlated (OR = 0.58,95%CI = 0.48 − 0.69). Hb also demonstrated a modest but significant positive association (OR = 1.03,95%CI = 1.01 − 1.04). Although RBC showed a positive trend (OR = 1.46,95%CI = 0.97 − 2.19), the result was not statistically significant (*P* = 0.072).

**Table 3:** Logistic regression analysis of OSAS parameters and blood indices associated with severe OSAS.

Variables	β	S.E	Z	*P*	OR (95%CI)
AHI	0.17	0.02	8.66	< 0.001	1.29 (1.14 ~ 1.44)
MinSaO_2_(%)	− 0.55	0.09	− 5.98	< 0.001	0.58 (0.48 ~ 0.69)
RBC(10^9^/L)	0.38	0.21	1.80	0.072	1.46 (0.97 ~ 2.19)
Hb(g/L)	0.03	0.01	3.47	< 0.001	1.03 (1.01 ~ 1.04)
HRR	1.06	0.16	6.52	< 0.001	1.09 (1.05 ~ 1.16)

**Abbreviations**: AHI, apnea hypopnea index; MinSaO2, minimum blood oxygen saturation; WBC, blood white blood cell; RBC, red blood cell; PLT, platelet; Hb, hemoglobin; HRR, hemoglobin to red blood cell distribution width ratio.

### Diagnostic performance and correlation analysis of HRR in severe OSAS

**Fig. 1:**
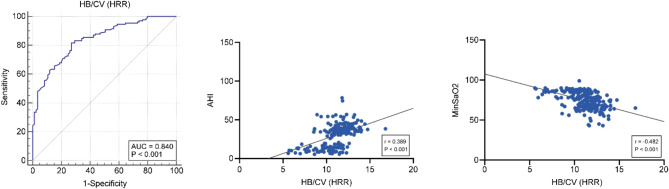
Predictive performance and correlations of HRR with severe OSAS parameters. (**A**) ROC curve analysis of HRR for predicting severe OSAS.(**B**) Positive correlation between HRR and AHI in severe OSAS patients.(**C**) Negative correlation between HRR and MinSaO₂ in severe OSAS patients.

The HRR demonstrated excellent predictive performance for severe OSAS. The area under the ROC curve (AUC) was 0.84 (95% CI: 0.784–0.886), with a standard error of 0.0265. This result was statistically significant (*P* < 0.0001). The optimal diagnostic cut-off value for HRR was > 11, which yielded a sensitivity of 81.68% and a specificity of 72.94%(Fig. 1**A)**. The HRR exhibited significant correlations with both the AHI and the MinSaO₂, highlighting its potential utility as a diagnostic marker for severe OSAS. Specifically, the correlation analysis revealed a positive correlation between HRR and AHI, with a correlation coefficient of *r* = 0.389 (*P* < 0.001), indicating that higher HRR values were associated with increased AHI levels(Fig. 1**B)**. In contrast, a negative correlation was observed between HRR and MinSaO₂, with a correlation coefficient of *r* = − 0.482 (*P* < 0.001), demonstrating that elevated HRR values were associated with lower minimum oxygen saturation((Fig. 1**C)**.These findings suggest that HRR is not only positively linked to the severity of apnea-hypopnea events, as reflected by AHI, but also inversely associated with the degree of oxygen desaturation, as indicated by MinSaO₂.

## **Discussion**

In recent years, OSAS has become a prevalent global chronic respiratory disease, particularly among middle-aged obese men, leading to repeated upper airway collapse during sleep. This results in apnea or hypoventilation, causing chronic intermittent hypoxia, hypoxemia, hypercapnia, and triggering inflammatory responses, oxidative stress, and abnormal vascular endothelial function. These processes can lead to systemic damage affecting multiple organs and systems, severely impacting quality of life and posing serious health risks^12^.While obesity is an independent risk factor for OSAS, the current study data show that the mean BMI is significantly higher in the severe OSAS group compared to the non-severe group. Obesity leads to fat deposition around the upper airway tissues, causing airway narrowing and collapse, which results in apnea or hypoventilation during sleep. Additionally, frequent nighttime awakenings and disruptions to normal sleep patterns can impair fat metabolism, creating a bidirectional relationship between obesity and OSAS^13^.

Studies have identified OSAS as an independent risk factor for conditions such as diabetes, cerebral infarction, and coronary heart disease, and it can also lead to various complications, worsening patient outcomes^14^. RDW is primarily used to diagnose and differentiate types of anemia, but recent studies show it is closely linked to chronic hypoxia and cerebral infarction^15^. Hemoglobin and RDW have also been associated with oxidative stress, inflammation, and the prognosis of certain malignancies^16–18^. The HRR is a newly introduced composite index, playing an important role in predicting the prognosis of cancers like esophageal squamous carcinoma, head and neck cancer, and lung cancer^19,20^. While HRR has proven predictive in malignancies and conditions like hepatitis B-related cirrhosis and rheumatoid arthritis, its role in OSAS has not been studied^21^.

In our study, HRR showed a statistically significant difference between the two patient groups. RDW, which reflects red blood cell variability, can be elevated in myocardial infarction patients due to inflammation, oxidative stress, and disrupted iron metabolism, leading to impaired erythropoiesis^22^. Previous studies have shown that RDW is elevated in OSAS patients and positively correlates with the AHI, suggesting it may indicate disease severity^23,24^. However, no significant difference in RDW was observed between the groups in this study, likely due to the small sample size, requiring further research. Some studies report higher Hb levels in OSAS patients, with a strong correlation between intermittent hypoxia and increased Hb^25–27^. However, other studies found no such increase in Hb in OSAS patients, though some researchers suggest Hb could be used to assess OSAS severity and predict complications^9^. In this study, Hb levels were significantly different between the two groups. Prolonged respiratory obstruction and apnea can reduce blood oxygen levels, triggering a compensatory rise in RBCs, which can also increase WBCs. However, no significant difference in WBC levels was found between the groups. We observed a more prominent difference in Hb levels compared to RBC counts between severe and non-severe OSAS groups. This may be attributed to compensatory increases in hemoglobin content per RBC under chronic intermittent hypoxia, as indicated by the significant changes in MCH and MCHC values.

Recent research on mean corpuscular volume (MCV) has proven useful in diagnosing anemia types and providing a basis for treatment^28^. Although correlations have been noted in conditions like rheumatoid arthritis, lupus, thalassemia, and chronic hepatitis B, there are few studies on MCV in OSAS patients, and this study found no significant difference between the groups^29,30^. In this study, we demonstrated that the HRR has excellent predictive performance for severe OSAS. The AUC of 0.84 (95% CI: 0.784–0.886) indicates that HRR is a reliable diagnostic marker for identifying severe OSAS, with a sensitivity of 81.68% and a specificity of 72.94% at an optimal diagnostic cut-off value of > 11. These findings are significant, as they highlight HRR as a practical and accessible tool for evaluating severe OSAS. Furthermore, we observed significant correlations between HRR, the AHI, and MinSaO₂, providing deeper insights into the role of HRR in OSAS pathophysiology. Specifically, the positive correlation between HRR and AHI (*r* = 0.389, *P* < 0.001) suggests that HRR increases in parallel with the severity of apnea-hypopnea events. This relationship may reflect the increased sympathetic activation triggered by recurrent apneic events, which leads to elevated heart rate variability. Sympathetic overactivity is well-documented in patients with OSAS and is a known consequence of repeated hypoxia and arousal from sleep. In contrast, HRR demonstrated a negative correlation with MinSaO₂ (*r* = − 0.482, *P*< 0.001), indicating that higher HRR values are associated with more severe oxygen desaturation. This inverse relationship likely arises because greater fluctuations in heart rate accompany significant hypoxemia, a hallmark of severe OSAS. As oxygen levels drop, compensatory mechanisms such as increased sympathetic activity aim to restore oxygen delivery to tissues, further emphasizing the link between HRR and hypoxic burden. The combined predictive value and correlations observed in our study support the potential of HRR as a non-invasive, objective biomarker for severe OSAS. Traditional diagnostic approaches, such as PSG, remain the gold standard but are often time-consuming, costly, and inaccessible for many patients^31^. In this context, HRR offers a simpler and more practical option for preliminary screening and risk stratification, particularly in resource-limited settings.

## Conclusion

However, it is important to acknowledge the limitations of our study. First, the sample size, while adequate, may not fully represent all OSAS phenotypes. Second, HRR may be influenced by other factors, such as age, cardiovascular comorbidities, and medications, which were not specifically addressed in this analysis. Future studies with larger cohorts and comprehensive adjustment for confounding variables are needed to confirm our findings and explore the underlying mechanisms further. HRR demonstrates strong predictive capability and significant correlations with both AHI and MinSaO₂, reinforcing its role as a valuable diagnostic indicator for severe OSAS. By offering insights into both respiratory event severity and the degree of hypoxemia, HRR holds promise for improving early detection and clinical management of OSAS.

## Electronic supplementary material

Below is the link to the electronic supplementary material.

Supplementary Material 1

## Data Availability

All data generated or analysed during this study are included in this published article(supplementary information files).

## Abbreviations

OSAS: obstructive sleep apnea syndrome
HRR: hemoglobin to red blood cell distribution width ratio
AHI: apnea hypopnea index
MinSaO_2_: minimum blood oxygen saturation
WBC: blood white blood cell
RBC: red blood cell
PLT: platelet
Hb: hemoglobin
RDW: red blood cell distribution width
HCT: hematocrit
MCV: mean corpuscular volume
MCH: mean corpuscular hemoglobin
MCHC: mean corpuscular hemoglobin concentration
ROC: receiver operating-characteristic
